# Ensuring no one is left behind: Urgent action required to address implementation challenges for NTD control and elimination

**DOI:** 10.1371/journal.pntd.0006426

**Published:** 2018-06-07

**Authors:** Alison Krentel, Margaret Gyapong, Olumide Ogundahunsi, Mary Amuyunzu-Nyamongo, Deborah A. McFarland

**Affiliations:** 1 Bruyère Research Institute, Ottawa, Canada; 2 Centre for Health Policy and Implementation Research, Institute for Health Research, University of Health and Allied Sciences, Ho, Ghana; 3 Research Capacity Strengthening, Special Programme for Research and Training in Tropical Diseases, WHO Geneva, Switzerland; 4 African Institute for Health and Development, Nairobi, Kenya; 5 Rollins School of Public Health, Emory University, Atlanta, Georgia, United States of America; The Hospital for Sick Children, CANADA

Since the ambitious goals to eliminate and control neglected tropical diseases (NTDs) were launched, the crucial role of partnerships has been emphasized as a pathway to ensure success. With multiple drug donations, we have a good supply of medicines necessary to eliminate parasites from the body; with donors, key funding for research and implementation; with researchers, the capability to create the evidence base for recommendations; with nongovernmental development organizations (NGDOs), support for implementation; and with national programs, the willingness and impetus to accomplish these ambitious goals. A country’s health system is almost always invoked as crucial to NTD implementation, with the claim that NTD programs contribute to the strengthening of health systems. Key partners, often missing at the table, are the endemic communities themselves. Yet we acknowledge that both communities and local health systems are the “backbone” of our programs.

Some key implementation questions frequently arise in NTD programs: sustaining the motivation of community drug distributors, appropriateness of timing of mass drug administration (MDA) activities, the coverage–compliance gap [1], social mobilization, human resource constraints in low- and middle-income countries, inefficient or weak health systems, multiple reporting requirements and different funding cycles from donors, and many more (see Fig 1). While these issues continue to plague our NTD community, we have not committed the necessary levels of research funds, expertise, or priority to adequately answer these questions. Often termed “social science questions,” these questions have been relegated to a category of research that is too difficult to conduct, too time-consuming, too costly, and seemingly less important than studies of drug efficacy or of the sensitivity of diagnostic tools.

**Fig 1:**
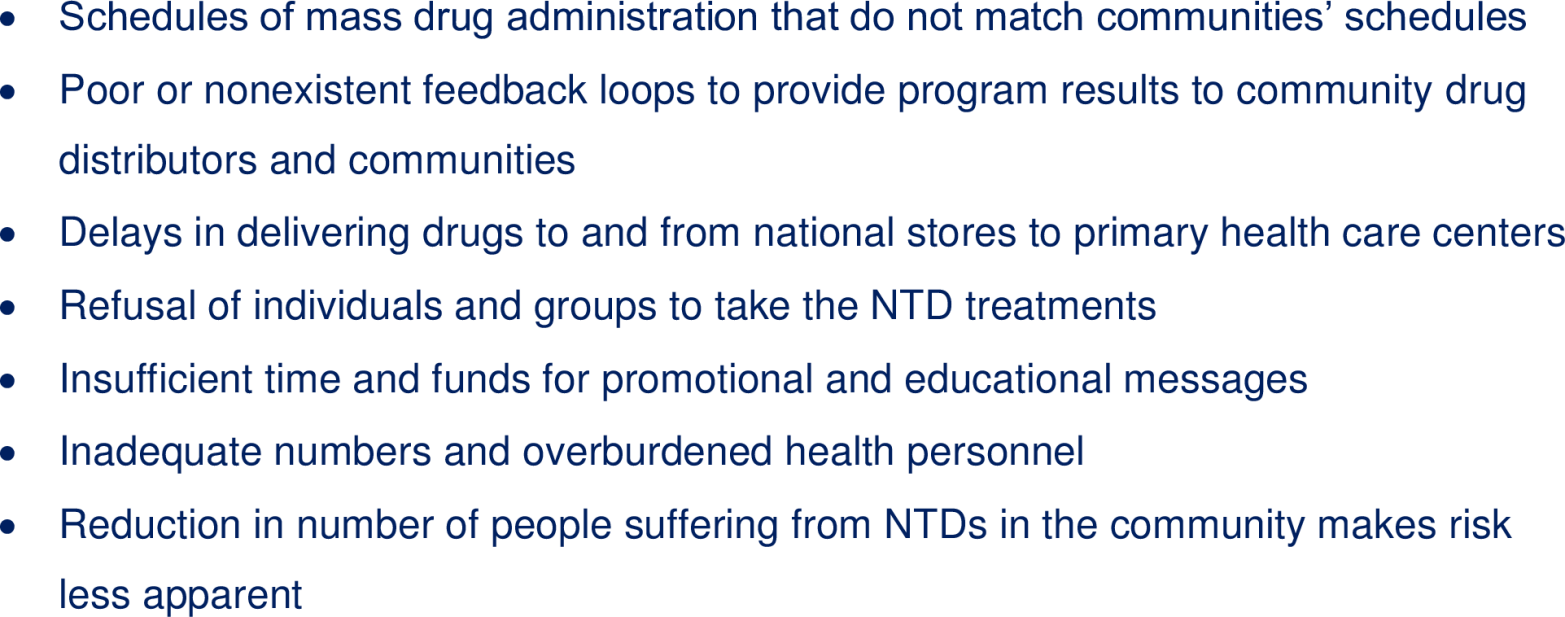
Examples of implementation barriers and bottlenecks inhibiting success in NTD programs.

We argue that it is time to bring these issues to the table and give them the attention they merit. We propose five principle areas for consideration by the NTD community. First, let’s rephrase these “social science” issues as implementation challenges that can be addressed by social science as well as epidemiological and other implementation research methods. Many disciplines such as anthropology, psychology, sociology, behavioral science, health economics, health services research, and other public health disciplines have the methods and tools to respond to these questions, which until now have been lumped together as “social science” questions. We need to acknowledge the strengths that each of these academic traditions can bring to our understanding of NTD elimination and control.

In addition, we need to ensure that the program implementers rather than the researchers are in the driver’s seat in the identification of the programmatic challenges and are valued members of the research team. Implemention research provides a framework for implementers and researchers to address implementation bottlenecks, identify optimal approaches for a particular setting, and promote the uptake of research findings. Ultimately, this process leads to improved healthcare and its delivery [2]. As we identify the implementation barriers and challenges that NTD programs face, we need to determine if they are global, regional, or country-specific issues. There is the urgent need for us to find out if these challenges are related to the community drug distributors, the community, the health system, the NTD program itself, or the funders of the program and try to address them in a more holistic way. When we spend time conducting implementation research to understand why barriers occur, we get the information we need to begin constructive dialogue with health personnel to understand the pertinent issues and to craft feasible solutions. Once interventions are put into place, we see evidence of improved programmatic outcomes [3,4,5].

Secondly, we should not shy away from the time and financial commitment that is needed to properly address these implementation questions. Getting the right answer today may ultimately save us time and money tomorrow. Within the context of the elimination program for lymphatic filariasis (LF), taking the time to understand why there are differences between reported coverage and actual compliance or why directly observed treatment is not implemented will provide the necessary information to adjust MDA, ensuring distributed drugs are consumed drugs. The time and expense of investigating these issues in a timely manner can translate into a savings of expensive additional MDA rounds if transmission assessment surveys fail. We do not develop new diagnostic tools overnight, and we should not expect to develop program diagnostic tools overnight, either. Both take time, resources, and periods of reiteration to optimize and determine the generalizability of the tool.

Thirdly, we should be more specific about the impact NTD programs have on health systems. There is a paucity of research into the interface between health systems and global health initiatives, like NTD control and elimination [6]. There is potential for enhancement in some elements of the health systems, such as drug procurement, health workforce, and community volunteers [6]. We know that NTD programs have mobilized and trained a large community workforce. These community drug distributors (CDDs) provide the critical link between the communities and health systems to ensure NTD program goals are met. Implementation research should focus on sustaining the gains of this workforce and help to develop key strategies to integrate it into other public health programs and structures [7,8]. We must also be cognizant that NTD programs can have negative effects on the health system [9]. For example, district health systems bear a considerable portion of the weight of an MDA campaign through coordination of training, social mobilization, drug distribution, supervision, mopping up, and reporting. These activities are implemented in addition to regular service delivery activities and sometimes with only limited supplementary budgets. Implementation research provides an appropriate framework from which to understand the effects of NTD programs on the health system, as the research is situated within the health system itself [2]. By conducting research in this manner, the outcome is more likely to lead to feasible, targeted recommendations with high potential to improve delivery of NTD activities in a sustained manner as well as influence policy revision and change. Within the current context of universal healthcare (UHC) and the Sustainable Development Goals (SDGs) [10], the formal inclusion of NTDs into health system planning and programming can ensure more equitable access to medications as well as continued management of the morbidities and disabilities resulting from prolonged infection. Including some NTD indicators in district health management information systems (DHIMSs) can provide sustainable monitoring.

Fourthly, we note that cost per treatment will likely increase as elimination programs begin to exhaust economies of scale and scope and move toward harder-to-reach populations. In the context of LF elimination, a recent WHO Weekly Epidemiological Report highlights some of these persistently challenging situations: cross-border areas, highly populated districts, and areas that have not yet begun MDA [11]. Activities and resource inputs will certainly change in the end game and will require that we are flexible, realistic, and adept at moving resources to the districts that may get left behind [12].

Lastly, as we dedicated ourselves to ensuring that no one is left behind in the Geneva commitment signed at the end of the NTD summit a year ago in April 2017 [13], let us mobilize our efforts to understand why some people, communities, and ethnic groups are left behind. As many national programs stop MDA in some districts, many others continue to have challenging areas where deeper understanding and direction are needed if these programs are to be successful. We acknowledge the systematic noncompliers as the potential reservoirs of infection [14], but we have done little to understand who these people are, why they have been left out, and what we need to do to reach them. Ignoring these kinds of implementation challenges risks maintaining them in perpetuity. We need the skill and creativity of many disciplines to respond to these challenges.

The 2020 goals are less than 30 months away. Now is the time to act. We are committed to those living in NTD-endemic communities and those working in local health systems, elevating their role as equal partners in our goal to effectively control and eliminate these diseases. Such respectful and equitable partnerships are a necessary pathway to success in 2020. Let us commit ourselves to advocating for the resources and political will to answer these implementation challenges correctly and sufficiently.

## Disclaimer

The views expressed in this article are those of the authors and do not represent the positions of their affiliated institutions.
